# Bayesian Hierarchical Model for Estimating Gene Expression Intensity Using Multiple Scanned Microarrays

**DOI:** 10.1155/2008/231950

**Published:** 2007-12-12

**Authors:** Rashi Gupta, Elja Arjas, Sangita Kulathinal, Andrew Thomas, Petri Auvinen

**Affiliations:** 1 Department of Mathematics and Statistics, University of Helsinki, P.O. Box 68, 00014 Helsinki, Finland; 2 Institute of Biotechnology, University of Helsinki, P.O.Box 56, 00014 Helsinki, Finland; 3 National Public Health Institute (KTL), Mannerheimintie 166, 00300 Helsinki, Finland

## Abstract

We propose a method for improving the quality of signal from DNA microarrays by using several scans at varying scanner sen-sitivities. A Bayesian latent intensity model is introduced for the analysis of such data. The method improves the accuracy at which expressions can be measured in all ranges and extends the dynamic range of measured gene expression at the high end. Our method is generic and can be applied to data from any organism, for imaging with any scanner that allows varying the laser power, and for extraction with any image analysis software. Results from a self-self hybridization data set illustrate an improved precision in the estimation of the expression of genes compared to what can be achieved by applying standard methods and using only a single scan.

## 1. Introduction

DNA microarray technology is used to study simultaneously the expression profile of a large number of distinct genes [1]. Several factors contribute to the accuracy with which these genes and their expressions (also referred here as intensities) can be determined. In particular, very low or very high in-tensities may lead to poor estimation of the ratio between the two samples and thus to an incorrect identification of differentially expressed genes. Low intensities tend to be noisy and lead to highly variable ratio estimates, whereas very high intensities are saturated from above and hence give biased results.

One of the objectives of microarray experiments is to identify a subset of genes that are differentially expressed be-tween the samples of interest. The relative intensity between the samples at a spot (also referred here as gene) is extracted by applying suitable image processing methods to the images produced by scanning the microarray slides on which the two samples have been hybridized. Errors occurring during image acquisition affect all further analyses and, therefore, the process of generation of these digital images is crucial. Photomultiplier tube (PMT), laser power (LP), and analog to digital converter (ADC), are the main components of an acquisition device, the scanner, which controls the for-mation of digital images. Each spot on the hybridized slide has fluorescent molecules corresponding to the two labeled samples and they emit photons upon excitation by a laser. The photons are converted into electrons by the PMT and the amount of current that eventually flows is directly proportional to the amount of incident light at the photocathode, unless saturation occurs [2]. Saturation oc-curs when the signal from a pixel exceeds the scanner's upper threshold of detection (, for a 16-bit computer storage system). This phenomenon is also called clipping, and it occurs when the ADC converter converts the electrons into a sequence of digital signals. This clipping effect renders the relation between the measured and the true intensities nonlinear in the upper range of intensities.

A single scanner setting will not be optimal for both weakly and highly expressed genes. The choice of the parameters (of a scanner) involves a trade-off between spots with a low intensity and spots that are saturated to some degree. Thus it seems reasonable to consider multiple scanning of the same microarray at different scanner sensitivities and estimate spot intensities from the combined data.

Not much work has been done so far on improving the data quality by using multiple scans and by adjusting for pixel censoring. Dudley et al. [3] increased the dynamic range of gene expression using a new method. They proposed to hybridize experimental and control samples against labeled oligos that would be complementary with respect to every microarray feature, rather than cohybridizing the samples. However, their method cannot be applied to experiments that follow the standard method of Schena et al. [1]. Khondoker et al. [4] presented a regression model based on a nonlinear relationship and involving both an additive and a multiplicative error terms to establish a link between the saturated and the true intensities, and used an approach based on maximum likelihood estimation to correct for saturation. Lyng et al. [5] recalculated the saturated signals using a set of unsaturated intensities from a second scan. Though they proposed a method to determine the unsaturated intensities, the main focus of their paper was on investigating the relationship between PMT voltages, spot intensities, and expression ratios for three commercial scanners of two different brands. Piepho et al. [6] suggested using a nonlinear latent regression model for correcting the bias caused by saturation and for combining data from multiple scans. Skibbe et al. [7] compared the number of differentially expressed genes when using approaches based on linear regression and when considering a union of sets of differentially expressed genes that had been identified by scans made by varying the PMT and laser power. They showed that the latter approach effectively identifies a subset of statistically significant genes that the former approach is unable to find.

Our approach towards improving quality of intensity measurements is based on first producing three images with different scanner sensitivities, and obtaining three different sets of expression values. We then apply a novel Bayesian latent intensity model, in which these different sets of expression values are used to estimate suitably calibrated true expressions of genes. The resulting estimates, treated in the form of respective (posterior) distributions, can be used in a higher-level analysis for identifying differentially expressed genes. The proposed approach is applicable to standard microarray methodology and to cDNA arrays. The method, however, cannot be applied to Affymetrix gene chips as the current Affymetrix technology does not allow multiple scanning. The method is generic and can be applied to data from any organism, imaging with any scanner that allows scanning at different laser powers, and extraction with any image analysis software.

## 2. Method

### 2.1. Data

In this study, we used cDNA microarrays containing 16 000 individual fragments printed in duplicate (produced in Turku Centre for Biotechnology, University of Turku, Finland). Our approach was tested on two experiments. The first experiment was designed to examine the effects of RhoG on HeLa cells by comparing expression profile of RhoG expressing cells versus control cells. This experiment was performed on two arrays (here called  and ). Each array had RNA from RhoG G12V labeled with Cy5, and control labeled with Cy3. The second experiment was a self-self hybridization experiment where RNA samples from T-Rex-HeLa cells (Invitrogen) transfected with a pcDNA4/TO vector were used. Details about sample preparation, RNA extraction, sample labeling and microarray hybridization for the two experiments can be requested from the authors.

### 2.2. Multiple Scanning

**Figure 1:**
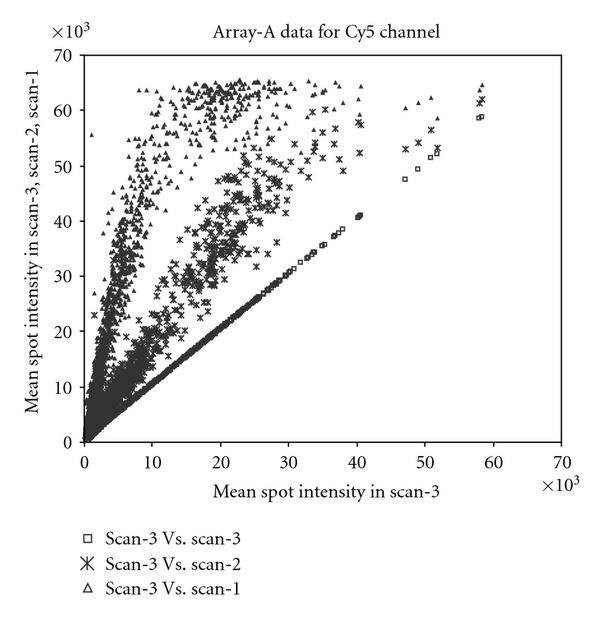
**Plot of multiple scans of array  for data from Cy5 channel**. The mean spot intensities from scan-3, scan-2, and scan-1 are plotted against scan-3. Saturation at the upper end of the intensities can be seen clearly. Very similar behavior was seen for the data from Cy3 channel.

The slides were scanned with ScanArray 5000 (GSI Lumonics) using an appropriate setting for samples labeled with Cy3 and Cy5. The line scan function of the scanner was used to equalize the intensities from Cy3 and Cy5 channels. A setting with the highest laser power was used to make the first scan (scan-1). Under this setting, because of high laser power, saturation occurs for spots with high RNA abundance, but the spots with low RNA abundance get captured accurately. This setting was chosen so that the background corrected intensity (foreground-background) from the spots with low RNA abundance was at least 200. Subsequent scans were performed with a lower laser power (reducing it by 10 units in each subsequent scan) for both channels but keeping the same PMT as in the first setting. With this lower laser power the number of spots with a saturated intensity decreases, whereas the number of spots with a low intensity (or below the threshold of 200 units) increases. Repeated scanning does not significantly damage fluorescence (data not shown), nor does lowering laser power affect the balance between the channels. Figure 1 shows the plot of multiple scans of array  and Table 1 shows the configuration of PMT and laser used to make multiple scans.

**Table 1:** The combinations of PMT and LP used to obtain multiple scans for array  and array .

Array	Scanner settings used for Cy3 (Cy5)			
	PMT Gain	Scan-1 (LP)	Scan-2 (LP)	Scan-3 (LP)
	85 (98)	100 (100)	90 (90)	80 (80)
	74 (85)	98 (100)	88 (90)	78 (80)

### 2.3. Quantification of Spot Intensities

Digital images were processed using GenePix Pro version 3.0 software (Axon Instruments, Inc., Foster City, Calif, USA. http://www.axon.com/GN _ GenePix4000.html). The automatic spot finding algorithm of GenePix was used to find spot boundaries and to calculate spot intensities. Spot fore-ground and background intensities for both channels were derived and background corrected intensities above 200 from all three scans were used for our study.

### 2.4. Latent Variable Model

Here we describe the model (on logarithmic (base e) scale) used for estimating a suitably calibrated "true underlying expression of a gene." We assume that each gene has an underlying signal, which cannot be measured directly. We call this underlying signal the *true latent intensity* of a gene and denote it on logarithmic (base e) scale by ,  where  is the number of spots used in the experiment. In the present experiment, each microarray chip was scanned at three different scanner settings. Let  index the scanner setting. For each array, the first scan (i.e., ) was made by using a scanner setting that would correspond to a situation in which only a single scan was per-formed.Therefore, these first scans form a natural basis for calibrating the corresponding true latent intensities . They can be expected to capture, without a downward bias caused by saturation, spots that do not have abundant levels of RNA. The second scan was then made by lowering the laser power from the level of the first scan, and the third scan by lowering it even more. The measured signals can then be expected to be correspondingly weaker, with the effect that also the degree of saturation in the measured signals on the highly expressed genes would be reduced, or even eliminated completely. Next, we assume that the second and the third scans are linked to  by simple functional relationships, respectively, by  and . Here  and  are unknown functions, which can naturally be assumed to be increasing and continuous, but which otherwise need to be estimated from the data. Since the range of gene expression data from the first scan was from  to , we decided to break the whole range into shorter intervals yet ensuring that there would be enough data points in each interval. We call these intervals  and assume a simple linear form for  and  in each of these intervals. In other words, we set(1)

where  is the length of the  interval.

Let  on logarithmic (base e) scale denote the observed intensity for spot  at settings , summarized over its pixels (corresponding to Cy3 or Cy5), where  and . In an ideal situation, the relationship be-tween the observed and the true intensities is expected to be proportional. However, as discussed above, the clipping caused by the ADC converter renders the relation to be nonlinear (Photomultiplier Tubes and Related Devices; Catalog June 2002, http://www.hamamatsu.com). If there were no measurement errors, we could write the observed intensity  in the form , whenever this intensity is below certain threshold (to be introduced below, and beyond which the intensities will be considered as right censored). Here, obviously  is the identity function. However, extraction of intensities of genes from a scanned microarray chip always involves some measurement errors. These errors might be minor, but can sometimes have a large effect on the observed intensity. Here we assume that the errors are modulated by the true signal level in a log-additive fashion. More exactly, we assume that for the observed intensities which are below this threshold, the relationship between observed and true intensitites can be expressed as(2)

where  is the error associated with spot  and scan .

Strictly speaking, right censoring at  (where 65 535 is the scanner's upper threshold of detection) is only appropriate in a pixel level model. In spot intensities, some degree of clipping takes place already well below this value. Piepho et al. [6] showed in their paper that spot saturation begins between 15 and 16 on log (base 2) scale, that is, somewhere between 32 768 and 65 535 on natural scale. The reason is that the signal from a spot is obtained by averaging the readings over the pixels belonging to the spot, and some of these pixels may be already saturated. As a result, and unlike in pixel level data, in spot level data there is no sharp threshold value beyond which saturation has an effect. Gupta et al. [8] provided data where, as could be expected, with increasing observed spot intensity also an increasing proportion of the pixel readings had reached their maximal value 65 535. At spot summary value of 60 000, most of the pixels comprising the spot were already saturated. However, although a pixel level model can be said to give a more truthful description of the saturation phenomenon as such, it cannot be applied in practice for analyzing pixel level data from arrays which typically con-tain several thousand spots. The reason is simply the computational cost involved, as each spot consists of 80–100 pixels. Here, instead of attempting to model the effect of saturation on observed spot intensities, we treat the high intensity readings, which are most affected by saturation, as right censored observations. We then compensate for the resulting loss of information by utilizing the measurements obtained with a lower laser power, finally combining, within the Bayesian framework, the information from all three scan measurements to obtain the posterior distribution of the true latent intensity. Right censored measurements are taken care of as a part of the same estimation process, by data augmentation, which effectively means that they are replaced by the corresponding conditional distributions. Applying such a process naturally still involves deciding on the level beyond which right censoring should take place. In the results reported here we considered signals which exceeded  (i.e., approximately 11) as right censored. Later, in Section 3, we consider the influence of the choice of the censoring threshold in some details.

To complete specification of the model, we assume errors  are independent and identically distributed Normal random variables with mean  and interval dependent variances , where ; . The interval dependent precision parameters (inverse of variances , , and , ) of errors , ,  were assigned gamma prior with parameters (). The true un-derlying latent intensities  are assigned Uniform prior distribution over the interval [, ], which is approximately []. The parameters (, ) are assigned Uniform distribution over ().

### 2.5. Bayesian Analysis

Several authors suggested Bayesian methods for analyzing microarray data [9–16]. Under the Bayesian paradigm, once the model is defined, statistical inferences can be expressed directly in terms of the conditional posterior probabilities conditioned on the observed data.

Our primary interest is in estimating the true underlying latent intensities , . We estimate them jointly with the parameters (, ), . Let  denote the parameters (including the latent intensities and the error variances) involved in the model. The joint distribution of  given the data  (, ; ) is given as(3)

A priori, the parameters , , and  are assumed to be independent. The numerical computations were done using Markov chain Monte Carlo (MCMC), where the sampling algorithm can be summarized as follows.

*Step 1*.

Specify initial values of , , , and of the augmented variables to be sampled when considering right censored 's.

*Step 2*.

Sample the latent intensities  from their conditional distribution.

*Step 3*.

Sample ,  from their conditional distribution.

*Step 4*.

Sample  from its conditional distribution.

*Step 5*.

Sample augmented 's from their conditional distri-butions.

*Step 6*.

Repeat step 2 to step 5 till sufficient samples are gen-erated.

The model was formulated in BUGS language and pa-rameter estimation was performed using WinBUGS [17]. WinBUGS is a free software and its newer versions can also run from within the statistical package . The current model runs in OpenBUGS version 2.01 on Intel Pentium processor 2.80 GHz with 1 GB RAM and takes approximately two hour to do 30 000 iterations using two chains in parallel. Convergence was monitored visually (i.e., by the mixing of two chains) and after a burn-in of 3000 iterations, two chains of 12 000 iterations each were generated to check the convergence of the parameter estimates under consideration. Thereafter, a sample of size 15 000 was generated to make inference.

## 3. Results

The approach described in this paper was tested on two real data sets described in Section 2.1 For the first experiment, samples were hybridized on two arrays  and  and each array was scanned three times at different scanner sensitivities (see Table 1). The same samples were hybridized on both arrays, but the scanner settings chosen for the two arrays were different as the experiments were performed independently on the two arrays.

To estimate , we need to first estimate the functional form of  and . Assuming piecewise linearity as described above, we divided the entire range (i.e., [(200), (65 535)] which is approximately [5.29, 11.09]) into intervals of length one. The break points chosen were (), leading to six intervals to be considered and with enough data in each interval. This division was based on the measurement reading from scan-1. The posterior median estimates of the parameters () over the six intervals together with the standard deviations of the posterior distributions are summarized in Table 2. The results do not support the assumption of an exact proportionality between the latent intensities as there are clear differences in the estimates of  and  over the six intervals. We also experimented with intervals of length 0.5 but faced convergence problems in MCMC as there were not enough data points in each interval. The choice of bin length will naturally have some impact on the results of the analysis; however, our experience from trying different intervals was that the differences were quite small. An attractive alternative would be to treat the break points as model parameters and then estimate them jointly with (), . This was not done here because of the additional computational burden that would result.

**Table 2:** Posterior median estimates of the parameters (, ) over the 6 intervals of data from the Cy3 dye.

Intensity range	Posterior median estimate of parameters (median ± sd) using data from Cy3 dye		
Lower limit	Upper limit		
5.29	6.29		
6.29	7.29		
7.29	8.29		
8.29	9.29		
9.29	10.29		
10.29	—		

**Figure 2:**
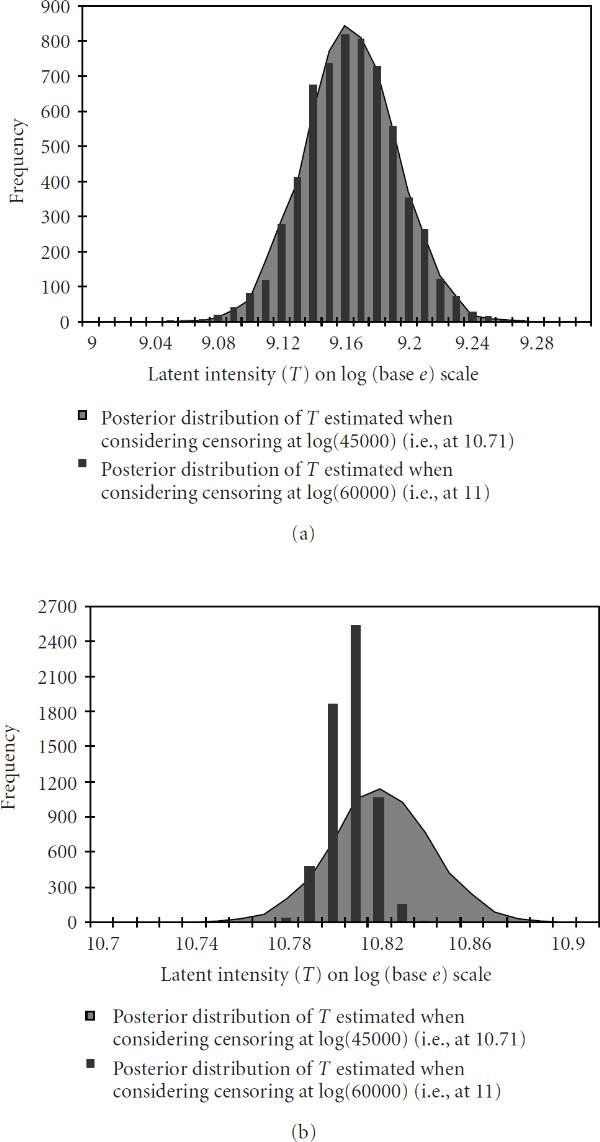
**Posterior distribution of the latent variable  for two genes obtained when considering censoring at  (i.e., at 10.71 and shown in grey) and when considering censoring at  (i.e., at 11 and shown in black bars)**. The measurements from "scan-1, scan-2, scan-3" are (a) (left-above) 9.15, 8.99, 8.61 and (b) (right-above) 10.84, 10.66, 10.23. (Different representations have been used to enhance visibility.)

In order to see how our estimation results would depend on the choice of threshold value beyond which the scan measurements are considered as right censored, we also experimented with values smaller than (60 000)11), considering then threshold values (45 000)10.71), (50 000)10.81), (55 000)10.91). Generally speaking, lowering this threshold value reduces the downward bias caused by saturation, but reduces also the precision of the posterior estimates. The differences are very small if all three scan readings are so low that none of them become right censored even when the lower of the two censoring thresholds is applied. This is illustrated by Figure 2(a), where we compare thresholds  and  and consider a spot for which the three scans were all well below . The very small differences between the two posterior distributions then are due to the differences in the estimates of the link functions  and . The situation changes somewhat if one of the three scan readings is high enough to be between  and , because it will be considered as right censored if  is used as the threshold, but remains uncensored if  is applied. This situation is illustrated in Figure 2(b), where we can already see a certain degree of tradeoff between bias and precision as was mentioned above. The difference between the two censoring schemes becomes even clearer if two of the three scan readings are between  and . Finally, if all three scan readings are right censored, the corresponding posterior will have only very low precision. Then the main message is that the true latent intensity from that spot is "very large".

**Figure 3:**
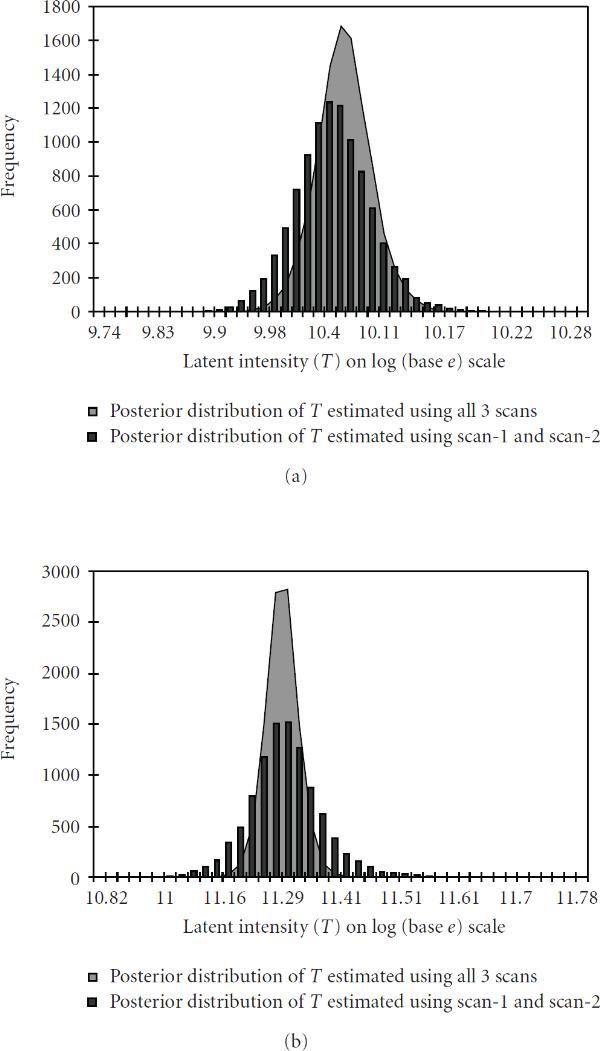
**Posterior distribution of the latent variable  for two genes obtained using all three scans (shown in grey) and using only two scans (scan-1 and scan-2, shown in black bars)**. The observation from "scan-1, scan-2, scan-3" for the genes are (a) (left-above) 10.51, 9.88, 9.08 and (b) (right-above) 11.01, 10.88, 10.50. (Different representations have been used to enhance the visibility.)

Comparison of the posterior distribution of the estimated latent variables (), for two randomly selected genes in different ranges, when using data from all three scans, and from only two scans (scan-1 and scan-2), is presented in Figure 3(a) and 3(b). These figures show that the spread of the posterior distribution when using data from all three scans (shown by grey area) is markedly smaller than when using data from two scans (shown in black bars). This corresponds to expectations since data extracted from scans made at three different sensitivity levels and then combined should provide more accurate inferences about the intensities compared to data from only a single or from two scans. Note that in Figure 3(b) the posterior is supported by values which are quite large as compared to the readings in scan-1, scan-2, and scan-3. The reason for this is that the measurement from scan-1 was above (60 000)11) and therefore treated as right censored, which led  to be estimated on the basis of measurements from scan-2, scan-3 and of sampled values of the augmented variable replacing the censored scan-1 observation. The posterior distributions of the latent variable  shown in Figure 3(b) are almost symmetric and well beyond (65 535)11.09).

**Figure 4:**
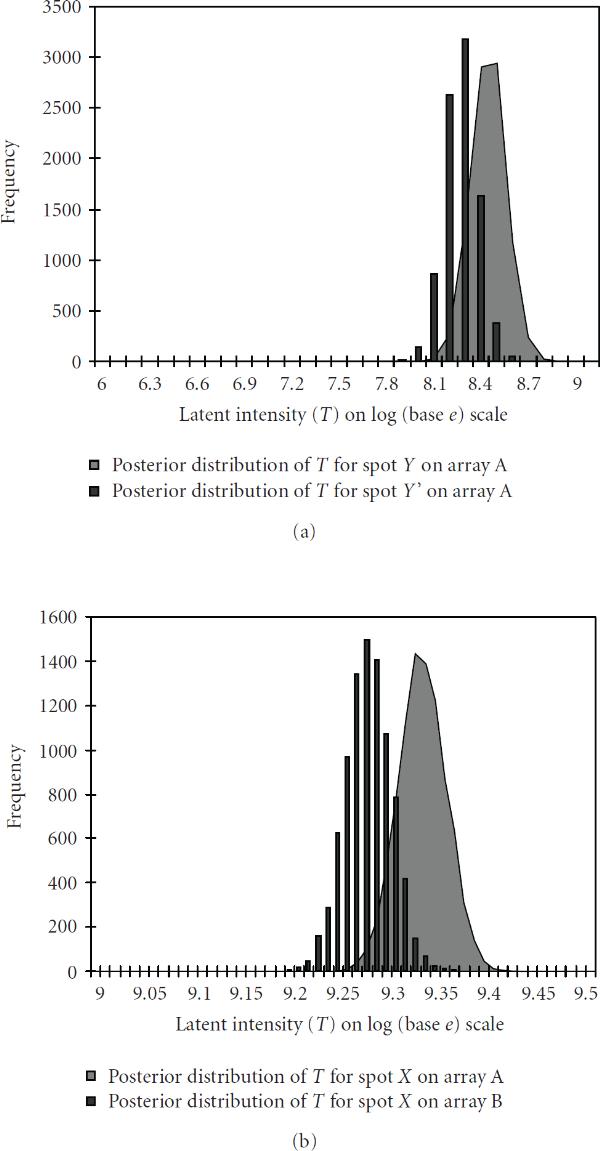
**Posterior distribution of true latent intensity for replicated spots on (a) (left-above) the same array A (b) (right-above) different arrays (array  and array ). The replicated spots  and  had "scan-1, scan-2, scan-3" measurements as 8.35, 8.26, 8.02 and 8.32, 7.94, 7.76, respectively, on array , and spot  had measurements 9.31, 9.23, 8.97 on array  and measurements 9.27, 9.17, 8.87 on array **. (Different representations have been used to enhance visibility.)

We also tested our method on replicated spots both within and across arrays. Since the data from two replicated spots are not directly comparable (without normalization) within an array due to the print-tip effect and across arrays due to the array effect [18,19], the corresponding latent intensity distributions often shifted away from each other and only had a small overlap as shown in Figure 4(a), 4(b). No normalization technique was applied for producing the plots.

**Figure 5:**
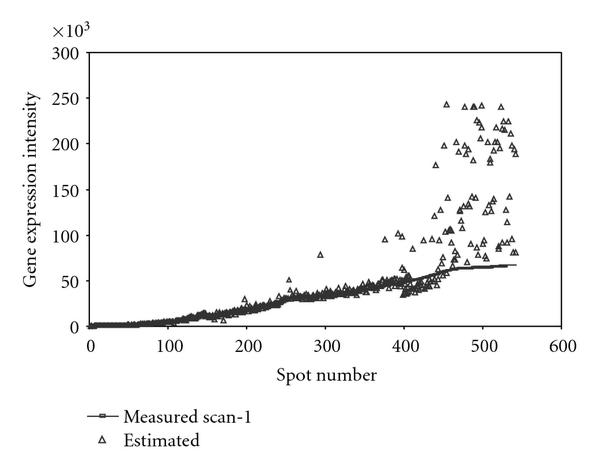
**This plot demonstrates the relashionship between the estimates of the latent intensities (on natural scale, for data from Cy3 channel) and the measurements from scan-1 over the range [200, 65 535] for 530 spots**. The intensities are sorted in an ascending order according to scan-1 reading.

The estimated posterior medians of the latent intensities (presented here on the natural scale) for data from Cy3 dye, along with the measurements from scan-1 (also on the natural scale) for 530 spots are displayed in Figure 5, It can be seen that the differences between the estimates and the scan-1 measurements are typically quite small when the scan-1 measurements are well below levels where saturation can be expected to have a systemic effect. However, the differences increase dramatically when the spot measurements approach levels around 60 000. Analogous results were obtained using data from Cy5 dye (data not shown).

**Figure 6:**
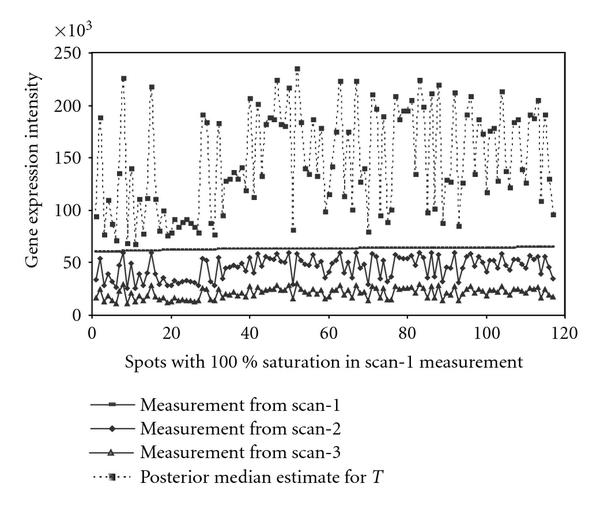
**This plot illustrates the dependence of the estimates of the latent intensities on the scan-2 and scan-3 readings in a situation in which the scan-1 readings were saturated**. 120 randomly selected genes with scan-1 measurement close to 65 535 are shown by a (nearly) horizontal line. Corresponding measurements from scan-2 and scan-3 are also plotted. The estimates of the latent intensities (posterior median) corresponding to these 120 spots are shown in dots and connected by dotted line. All measurements are on natural scale.

Figure 6 (presented on the natural scale) illustrates how the saturated measurements of 120 spots from scan-1 get estimated when using additional observations from their corresponding scan-2 and scan-3 measurements. In this figure, the scan-1 measurements are shown by a nearly horizontal line, close to the maximal value 65 535. Since any reading from scan-1 beyond 60 000 was treated as right censored, we can see that the corresponding estimates of the latent intensities are generally far beyond 65 535 and that they demonstrate the same pattern as observed in scan-2 and scan-3. Thus the measurements from scan-2 and scan-3 help in inferring the true signal when the corresponding scan-1 reading becomes saturated.

**Figure 7:**
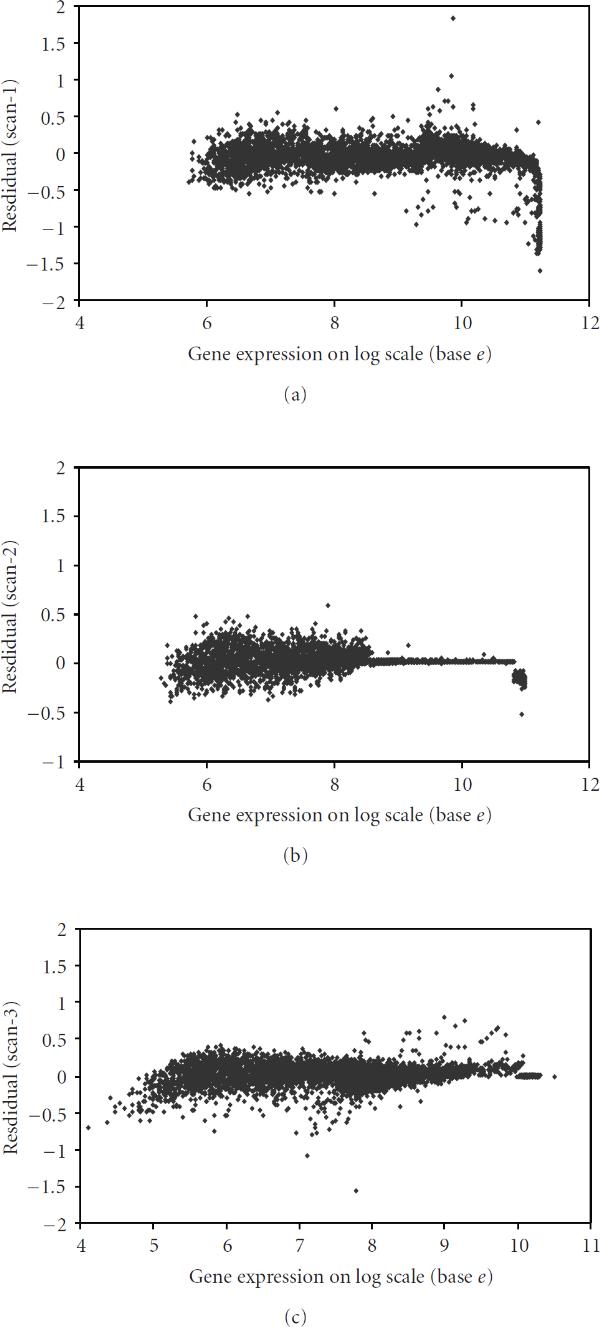
**Estimated residuals (= measured values - corresponding posterior median) from the empirical data plotted against the rank of the estimated gene expression for (a) (left-above) scan-1, (b) (middle-above) scan-2, and (c) (right-above) scan-3**.

We validated the fit of the model by plotting the re-siduals arising from each of the three scans as displayed in Figure 7(a), 7(b), 7(c). This was done quite easily in WinBUGS, by defining the residual for each of the spots as the difference of the (logarithmic) observed and the estimated value (in the sense of posterior median). It can be seen from the plot (Figure 7(a)) corresponding to the first scan that the residuals are generally quite small but that there are some, at the high end of the range of expressions, that have relatively large (negative) values. The reason for such large residuals is that, as the observed scan-1 values get close to saturation, the estimated latent values become generally bigger than the observed scan-1 readings. This is precisely what our method was designed to do: correct for the bias caused by saturation. This same phenomenon can be seen more clearly from Figure 6, where we have plotted the estimate of latent intensity, together with measurements from scan-2 and scan-3, for all genes for which scan-1 reading was completely saturated. To sum up, residuals over the three scans are small implying a good fit of the real data to our model.

**Figure 8:**
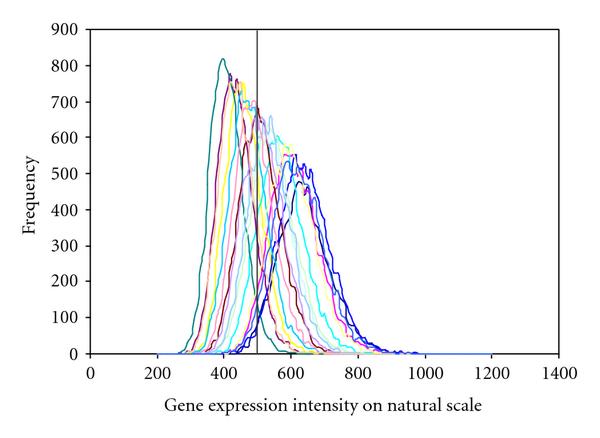
**These plots illustrate the sample variability of the posterior distributions, considering (latent) gene expression intensities and in each case 17 simulated samples**. The true expression value (on natural scale) is shown with a vertical bar.

A different way of illustrating the performance of our method can be provided by using simulated data (from the same model). For each data set generated by our model, and for each gene, we obtain the posterior distribution of the "true" expression level (known from the simulation), and can thereby quantify the degree of uncertainty in the estimation of that value (then pretending, for a moment, that it was unknown). This is illustrated in Figure 8 for a particular gene.

**Figure 9:**
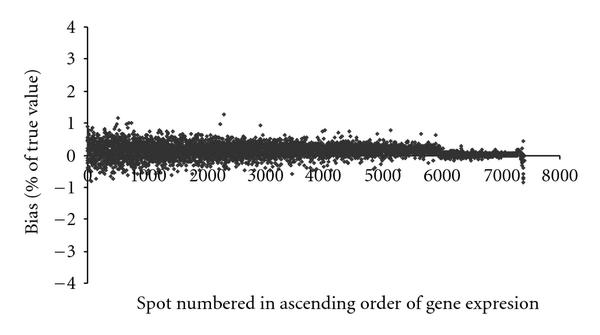
**Estimated percentage of bias plotted against the spot numbers, based on a simulation experiment**.

Finally, to also consider simulations where the data had been generated by a different model, we generated 100 datasets from model (2) of Khondoker et al. [4] by using the "true" values denoted there by  and the parameter values as specified for array-2 data in their Table 1. In order to assess whether our method would introduce systematic bias into the estimates, following the setting of Figure 4 of Khondoker [4]), we computed, for each gene, the arithmetic mean of the 100 estimated expression levels (in our case, determined as posterior means) and then compared it with the corresponding "true" value (). The differences, computed in each case as a percentage of the corresponding true value, are plotted in Figure 9 against the rank of true values. In order to provide a direct comparison with Figure 4 of Khondoker [4], we calibrated our model according to scan-3 (corresponding to the lowest intensities), rather than scan-1 as was done above. Again, no significant systematic biases can be traced from these plots.

**Figure 10:**
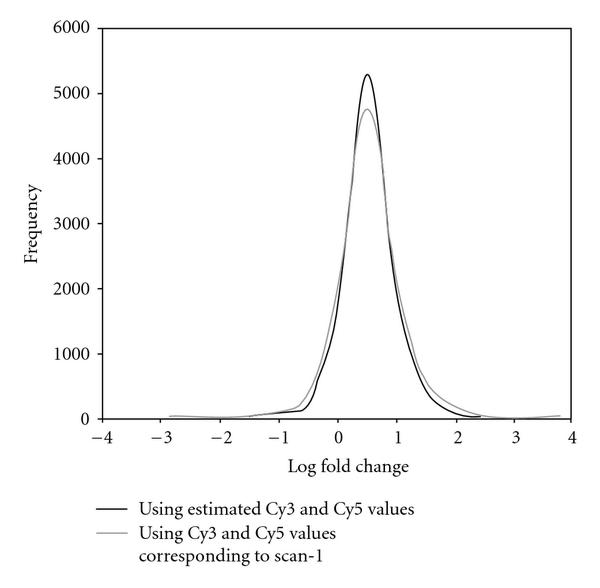
**Comparison of the histograms of the log fold change corresponding to the scan-1 data (shown in grey) and the estimated posterior median (Cy3, Cy5) (shown in black)**.

In our work the correction for the two dyes was done separately, as the labeling efficiencies and fluorescent properties of the two dyes are different. The existence of systematic differences between the two dyes has been documented in the literature, and they have been treated separately by [4,20,21] while correcting for signal saturation. The separate correction for the two dyes eventually affects the log ratios (also known as log-fold change) between the two channels. We plotted in Figure 10 the log ratios (= difference of the posterior median estimates of ) corresponding to the two channels together with the log-ratios (= difference of difference of the observed intensities corresponding to scan-1 , on which commonly all higher level inferences are based) for the self-self hybridization data set. Spots with the number of foreground pixels less than 80 pixels and spots flagged by GenePix as not reliable (flagged as , ) were excluded from the analysis. The histogram of the log ratios corresponding to scan-1 data is shown in grey and that of the log ratios corresponding to estimated posterior medians (Cy3, Cy5) in black in Figure 10. The data were not normalized and, therefore, the two histograms are not centered at 0. The  axis in Figure 10 is in thousands and thus even a small difference between the two histograms leads to hundreds of spots being potentially classified differently. Therefore, if differentially expressed genes were to be identified by any cutoff value of the log ratio, more genes would be declared as statistically significant when using scan-1 data than when using our approach. Since in this case there are no true differentially expressed genes, the former method would result in a larger number of false positives. This supports our hypothesis that generally more precise inferences can be drawn when using the present method, which combines inferences from three scans.

## 4. Conclusions

Here our focus has been on the systematic bias in the intensity measurements caused by intrinsic scanner noise at the lower end and pixel censoring at the upper end. These two problems cannot be handled under a single scanner setting. Moreover, guidelines are not available for choosing optimal scanner settings to address both of these issues. Therefore, it seems reasonable to do several scans on every array, some at relatively lower sensitivities (ensuring no censoring at the upper end) and others at higher sensitivity levels (to capture weakly expressed genes), and ultimately combine the information to get improved gene expression measurements at all ranges. More scans can easily be accommodated in the model but there are practical limitations like degradation of the dye and the time required for scanning. Keeping these points in mind, three scans seem to be a good compromise.

The proposed method has advantages at three levels. First, modeling under the Bayesian framework allows for missing data estimation by sampling randomly from the corresponding posterior predictive distribution. Second, it allows for joint estimation of a large number of model parameters and latent variables. Usually for analyzing microarray data, the statistical methods are applied in a sequential manner with the output of each step in the analysis serving as the input for the next. Under the sequential approach, the uncertainties in the conclusions from any earlier step make the subsequent steps dependent on the particular choice of the method and the resulting point estimate that is then used. In our model, such uncertainties are accounted for in a systematic manner as we work with distributions of all the unknown parameters, including the latent expression of the genes being considered. A third aspect of our method is that it opens up the possibility of extending the current model to accommodate features of normalization and identification of differentially expressed genes in an integrated model, which first improves the overall signal and then identifies differentially expressed genes by using such improved signals. Realization of the integrated model is in principle a straightforward modification to the model proposed here, by adding further layers to the present hierarchical model. Such additional layers would then account for between-array variations, within-array variations, and dye swap, and allow for comparing and combing data from multiple arrays. We are currently working towards such an integrated model.

## Appendix

See Algorithm 1.

**Algorithm 1:** Code written in BUGS language.

model

cut1<- 6.29

cut2<- 7.29

cut3<- 8.29

cut4<- 9.29

cut5<- 10.29

for(i in 1 : N) 

T[i]<- 

muYe1[i]  dunif(0, 15)

class[i]<- 1 + step(logye1[i] - cut1) + step(logye1[i] - cut2) + step(logye1[i] - cut3) + step(logye1[i] - cut4) + step(logye1[i] - cut5)

muYe2[i]<- A[i]+B[i]+C[i]

A[i]<- (b[1]  muYe1[i])  step(cut1-logye1[i]) + (b[1] + (b[2]  (muYe1[i]-1)))  step(cut2-logye1[i])  step(logye1[i]-cut1) +

(b[1] + b[2] + (b[3]  (muYe1[i]-2)))  step(cut3-logye1[i])  step(logye1[i]-cut2)

B[i]-(b[1] + b[2] + b[3] + (b[4]  (muYe1[i]-3)))  step(cut4-logye1[i])  step(logye1[i]-cut3) + (b[1] + b[2] + b[3] + b[4] +

 (b[5]  (muYe1[i]-4)))  step(cut5-logye1[i])  step(logye1[i]-cut4)

C[i]-(b[1] + b[2] + b[3] + b[4] + b[5] + (b[6]  (muYe1[i]-5)))  step(logye1[i]-cut5)

muYe3[i] -D[i]+E[i]+F[i]

D[i]-(d[1]  muYe1[i])  step(cut1-logye1[i]) + (d[1] + (d[2]  (muYe1[i]-1)))  step(cut2-logye1[i])  step(logye1[i]-cut1) +

(d[1] + d[2] + (d[3]  (muYe1[i]-2)))  step(cut3-logye1[i])  step(logye1[i]-cut2)

E[i]-(d[1] + d[2] + d[3] + (d[4]  (muYe1[i]-3)))  step(cut4-logye1[i])  step(logye1[i]-cut3) + (d[1] + d[2] + d[3] + d[4] +

(d[5]  (muYe1[i]-4)))  step(cut5-logye1[i])  step(logye1[i]-cut4)

F[i]-(d[1] + d[2] + d[3] + d[4] + d[5] + (d[6]  (muYe1[i]-5)))  step(logye1[i]-cut5)

logye1[i] ~ dnorm(muYe1[i], tau1[class[i]]) I(logye1cen[i], )

logye2[i] ~ dnorm(muYe2[i], tau2[class[i]]) I(logye2cen[i], )

logye3[i] ~ dnorm(muYe3[i], tau3[class[i]]) I(logye3cen[i], )

for(j in 1 : nClass)

tau1[j]  dgamma(0.001, 0.001)

sigma1[j]- 1 / sqrt(tau1[j])

tau2[j]  dgamma(0.001, 0.001)

sigma2[j]- 1 / sqrt(tau2[j])

tau3[j]  dgamma(0.001, 0.001)

sigma3[j]- 1 / sqrt(tau3[j])

b[j] ~ dunif(0,5)

d[j] ~ dunif(0,5)

for(i in 1 : N)

residual1[i]-Ye1[i]-muYe1[i]

residual2[i]-Ye2[i]-muYe2[i]

residual3[i]-Ye3[i]-muYe3[i]

where N is the number of genes, logye1, logye2, logye3 are the measurements from the three scans on logarithmic scale,

I(logye1cen[i], ), I(logye2cen[i], ), I(logye3cen[i], ) were used to specify the lower bound for the censored measurements

from the three scans.
